# Amputation in the Course of Diabetes

**Published:** 1902-04-12

**Authors:** 


### AMPUTATION IN THE COURSE OF DIABETES.

It was long held that the existence of diabetes should absolutely preclude all operations except such as were required for the immediate preservation of life. Under modern methods, however, many operations on diabetic subjects have been successful which in former times would have hardly been regarded as permissible. Mr. G. W. Spencer[1] has gone a stage further, and has reported a case in which not only was he able to operate in the course of diabetes without evil consequences, but in which, apparently as the result of the operation, the diabetes itself passed away. The patient was a woman aged 61, who had been a chronic diabetic for perhaps a dozen years. About the beginning of October she first noticed a small crack at the side of the nail of the second toe of the right foot, whence inflammation spread over the foot, pus escaping from several openings. Extensive necrosis was found in the foot and no pulsation could be discovered below Scarpa's triangle on that side. On December 17th amputation of the right limb just below the middle of the thigh was performed. A dissection showed general necrosis of the bones of the foot with infiltration of the soft parts by suppuration. Sugar continued to be present in the urine until January 10th, after which none was found, and she left the hospital on March 8th.

[1] Lancet, March 22.

